# Arbuscular Mycorrhiza and Antagonistic Microbial Consortia Reduce Phytopathogenic Pressure and Improve Rhizosphere Functioning of Sugar Beet Under Short-Rotation Cropping Systems

**DOI:** 10.3390/plants15101529

**Published:** 2026-05-16

**Authors:** Dmytro Kyselov, Svitlana Kalenska, Andrii Kyselov, Mykhailo Chonka, Bohdan Mazurenko

**Affiliations:** 1 Private Enterprise “Zakhidnyi Buh”, 39 Yunosti Avenue, Pavliv Village, Sheptytskyi District, 80250 Lviv, Ukraine; dmytro.kyselov@zahbug.com.ua (D.K.); andrii.kyselov@zahbug.com.ua (A.K.); myxailo.chonka@zahbug.com.ua (M.C.); 2 Department of Plant Science, National University of Life and Environmental Sciences of Ukraine, 15 Heroiv Oborony Str., 03041 Kyiv, Ukraine; kalenskaya@nubip.edu.ua

**Keywords:** root diseases, biological control, microbial inoculation, dehydrogenase activity, agroecosystem resilience

## Abstract

Short-rotation sugar beet (*Beta vulgaris* L.) cultivation in the Western Forest-Steppe of Ukraine is often accompanied by increased phytopathogenic pressure and impaired rhizosphere functioning, creating a need for biological tools to stabilize the plant–soil system. This study evaluated the effects of arbuscular mycorrhiza and an antagonistic microbial consortium on pathogen pressure, rhizosphere activity, yield, and technological quality of sugar beet under different crop rotations. Field experiments were conducted in 2023–2025 using a three-factor design that included rotation, mycorrhizal inoculation, and microbial inoculation. The highest phytopathogenic pressure was recorded in the maize–soybean–sugar beet rotation, where the cumulative frequency of dominant pathogens reached 94.0% and the root rot severity index in the control was 28.6%. Arbuscular mycorrhiza reduced disease development by 14.6–16.4%, whereas the antagonistic consortium reduced it by 25.6–27.9% relative to the control. Their combined application was most effective, decreasing root rot severity to 9.6–17.1% and increasing root colonization, available phosphorus, and dehydrogenase activity in the rhizosphere. The highest yield (80.5 t/ha) and sugar content (18.5%) were obtained in the soybean–winter wheat–sugar beet rotation under combined inoculation. AMF can improve phosphorus acquisition and mycorrhiza-induced tolerance, whereas antagonistic fungi can directly suppress soil-borne pathogens through competition, antibiosis, and mycoparasitism, their combined use may provide complementary protection in disease-conducive rotations. Overall, integrating arbuscular mycorrhiza with antagonistic microorganisms is a promising approach for reducing pathogen pressure and improving sugar beet performance in short-rotation systems.

## 1. Introduction

Sugar beet is one of the major industrial crops of the temperate zone and remains important not only for sugar production, but also for the bioeconomy, livestock feeding, and the processing industry. In modern agroecosystems, the stability of sugar beet productivity depends on the ability of the production system to maintain functional balance under climate variability and elevated phytosanitary pressure use [1,2]. In regions with a high concentration of sugar beet production, shortened crop rotations are becoming a major phytosanitary concern because repeated cultivation may increase the risk of soil-borne diseases and reduce the technological quality of harvested roots [1,2].

One of the main consequences of shortened rotations is the accumulation of soil-borne pathogens, including *Rhizoctonia solani*, *Aphanomyces cochlioides*, and *Fusarium* spp., which are commonly associated with root rot and reduced crop productivity [3,4]. The negative effects of short rotations are not limited to pathogen build-up. They may also involve disruption of rhizosphere functioning, reduced microbial suppressiveness, and weaker plant–soil–microbiota interactions, thereby increasing crop susceptibility to biotic stress [2,5].

Among such biological agents, arbuscular mycorrhizal fungi (AMF) are of particular interest because they improve the uptake of phosphorus and other relatively immobile nutrients, modify the root–soil interface, and may alter the composition of rhizosphere-associated microbial communities [6,7]. In addition to their nutritional role, AMF may contribute to plant defense through mechanisms associated with mycorrhiza-induced resistance. These effects may vary according to soil conditions, crop rotation, plant genotype, and the composition of the surrounding microbial community [6,7].

Antagonistic fungi, particularly *Trichoderma*-based consortia, represent another biological approach for regulating pathogen pressure in the rhizosphere. Their activity may involve direct antagonism against soil-borne pathogens, competition for ecological niches, stimulation of plant growth, and enhancement of soil enzymatic activity [8,9,10]. In sugar beet, such effects are especially relevant because resistance to root diseases is shaped not only by host genotype, but also by microbiome-mediated mechanisms that influence plant tolerance and rhizosphere stability.

Despite the rapid development of plant–soil–microbe research, comprehensive field studies in sugar beet are still limited, particularly those evaluating rotational load, the contribution of AMF, the effects of antagonistic microbial consortia, and their potential synergistic role in reducing phytopathogenic pressure, stabilizing rhizosphere functioning, improving productivity, and enhancing root quality [11,12]. This knowledge gap is especially relevant in the Western Forest-Steppe of Ukraine, where shortened rotations, high spatial concentration of sugar beet, and heterogeneous soil conditions intensify phytosanitary risks and complicate the maintenance of stable crop health [3,4].

Previous studies have described the effects of crop sequence on sugar beet yield formation, the role of preceding crops in Rhizoctonia inoculum carryover, and the ability of beneficial microorganisms to reshape rhizosphere communities [3,10,13]. However, most available evidence considers these components separately. It remains insufficiently known whether AMF and antagonistic fungi are compatible under field conditions and whether their co-application can simultaneously reduce pathogen occurrence, strengthen rhizosphere functioning, and improve yield and technological quality in short-rotation sugar beet systems. Short-rotation sugar beet is also insufficiently studied from this integrated perspective because many previous studies focused either on yield response, individual pathogens, or continuous cropping effects [14], rather than on the combined rotation–microbiome–disease–quality pathway.

Therefore, the aim of this study was to evaluate the effects of arbuscular mycorrhiza and an antagonistic microbial consortium, applied separately and in combination, on phytopathogenic pressure, rhizosphere functioning, yield, and technological quality of sugar beet under different levels of rotational load. We tested the hypotheses that maize-containing short rotations would increase pathogen frequency and root rot severity by promoting pathogen carryover and weakening rhizosphere suppressiveness, whereas combined AMF + antagonistic inoculation would provide stronger protection than either component alone by integrating AMF-mediated nutrient acquisition and defense priming with antagonist-mediated pathogen suppression.

## 2. Results

### 2.1. Pathogen Occurrence Under Different Rotation Backgrounds

The structure of the pathogen complex depended strongly on the rotation background (Table 1). Across all rotations, the dominant pathogens were *Fusarium oxysporum*, *F. solani*, *Phoma betae*, *Rhizoctonia solani*, and *Botrytis cinerea*. The highest cumulative frequency of dominant pathogens was recorded in the maize → soybean → sugar beet rotation (94.0 ± 1.7%), which significantly exceeded the values observed in the other rotations. The lowest infection background was found in the soybean → winter wheat → sugar beet rotation (68.0 ± 1.3%). Among individual pathogens, the rotation effect was most evident for *F. oxysporum* and *R. solani*, indicating that these taxa were the most responsive to changes in preceding crops. Their higher occurrence in maize-containing rotations suggests that maize may contribute to pathogen carryover or create a rhizosphere environment more favorable for the persistence of soil-borne fungi. The increase in *P. betae* and *F. solani* further indicates that the effect of maize was not limited to a single pathogen, but reflected a broader shift toward a more disease-conducive pathogen complex. In contrast, *B. cinerea* showed little variation among rotations, suggesting that its occurrence was less dependent on crop sequence and probably more influenced by general environmental conditions during the growing season.

The higher pathogen frequency in maize-containing rotations may be explained by greater inoculum carryover and by a less favorable break-crop effect before sugar beet. Maize can support or maintain several soil-borne fungal groups, especially Rhizoctonia-associated inoculum, and its residue decomposition may prolong the period during which pathogen propagules persist in the upper soil layer. Therefore, the increase observed in these rotations should be interpreted not only as a numerical rise in individual taxa, but as a shift toward a more disease-conducive rhizosphere background before sugar beet establishment.

### 2.2. Root Rot Development in Response to AMF and Antagonistic Inoculation

The root rot severity index confirmed the strong effect of rotation background on crop health (Table 2). In the control treatment, the highest disease level was recorded in the maize → soybean → sugar beet rotation (28.6 ± 0.8%), whereas the lowest was observed in the soybean → winter wheat → sugar beet rotation (16.5 ± 0.5%). This pattern indicates that the suppressive or conducive character of the preceding crop sequence strongly affected the initial phytosanitary status of sugar beet. Maize-containing rotations created a more disease-conducive background, most likely because of greater pathogen carryover and weaker biological suppression in the rhizosphere.

AMF inoculation consistently reduced disease development in all rotations, but its effect was moderate compared with the antagonistic consortium. Relative to the corresponding controls, the root rot index decreased by 14.6–16.4% depends on crop rotation. The antagonistic consortium showed a stronger effect, reducing disease development by 25.6–27.9%. In contrast to AMF, the antagonistic treatment was expected to act more directly on the pathogen complex through competition for space and nutrients, mycoparasitism, antibiosis, and stimulation of plant responses. Therefore, the higher effectiveness of the antagonist treatment indicates a stronger direct biocontrol component in the suppression of root rot.

The combined AMF + antagonistic treatment was the most effective across all crop rotations. Compared with the control, combined inoculation reduced root rot severity by 37.8–41.8% (9.6–17.1% in absolute values). Thus, the combined treatment was about 2.5 times more effective than AMF alone and about 1.5 times more effective than the antagonistic consortium alone in terms of relative disease reduction. The lowest disease level was recorded in the soybean → winter wheat → sugar beet rotation under combined inoculation, whereas even in the most unfavorable maize → soybean → sugar beet rotation, the combined treatment substantially reduced disease severity compared with the control and single-inoculant treatments.

These results indicate that root rot suppression was not caused by a single mechanism, but by complementary interactions within the AMF ↔ plant ↔ pathogen ↔ antagonist system. AMF improved host tolerance and rhizosphere functioning through nutrient acquisition and defense priming, while the antagonistic consortium directly constrained pathogen development. The plant mediated these interactions through root exudation, nutrient demand, and tolerance activation. Therefore, the combined treatment provided both indirect plant-mediated protection and direct pathogen suppression, explaining its consistently higher effectiveness across rotation backgrounds.

### 2.3. AMF Root Colonization and Rhizosphere Functioning

The biological treatments significantly affected AMF root colonization and rhizosphere activity (Table 3). In the control, natural AMF colonization of roots was 12.8 ± 0.5%, whereas AMF inoculation increased this value to 35.6 ± 0.9%. Under combined inoculation with AMF and antagonists, colonization reached 37.4 ± 1.0%, indicating that the antagonistic consortium did not suppress mycorrhizal establishment.

The content of available phosphorus in the rhizosphere soil also increased under biological treatments. In the control, it was 13.2 ± 0.3 mg/100 g soil, compared with 15.0 ± 0.4 mg/100 g under AMF and 15.8 ± 0.4 mg/100 g under AMF + antagonists. Antagonists applied alone maintained phosphorus availability at 13.9 ± 0.3 mg/100 g soil.

A similar trend was observed for rhizosphere enzymatic activity. Dehydrogenase activity increased from 29.1 ± 0.7 mg TPF/kg/24 h in the control to 32.0 ± 0.8 mg TPF/kg/24 h under AMF, 33.4 ± 0.8 mg TPF/kg/24 h under antagonists, and 35.8 ± 0.9 mg TPF/kg/24 h under combined inoculation. Phosphatase activity was also highest under AMF + antagonists, reaching 5.9 ± 0.1 mg P_2_O_5_/100 g/h, compared with 4.9 ± 0.1 mg P_2_O_5_/100 g/h in the control.

These patterns indicate that AMF and antagonists affected different but complementary components of rhizosphere functioning. AMF most likely increased phosphorus availability by expanding the effective absorbing surface of the root system and stimulating phosphatase-mediated P mobilization, whereas the antagonistic consortium mainly increased overall microbial oxidative metabolism, as reflected by dehydrogenase activity. AMF alone and in combination with antagonists formed the highest group for root colonization, confirming that the antagonists did not inhibit mycorrhizal establishment, while the combined treatment was significantly superior for available P, dehydrogenase activity, and phosphatase activity.

### 2.4. Effect of the Antagonistic Consortium on the Rhizosphere Pathogen Complex

The antagonistic consortium significantly reduced the frequency of all major pathogens detected in the rhizosphere (Table 4). The frequency of *F. oxysporum* declined from 29.0 ± 0.8% to 23.0 ± 0.7%, and that of *F. solani* from 17.0 ± 0.5% to 13.0 ± 0.4%. The strongest absolute reductions were observed for *Phoma betae*, *Rhizoctonia solani*, and *Botrytis cinerea*, whose frequencies decreased from 15.0 ± 0.5% to 10.0 ± 0.3%, from 12.0 ± 0.4% to 8.0 ± 0.3%, and from 9.0 ± 0.3% to 6.0 ± 0.2%, respectively.

Relative reduction analysis showed that the antagonistic consortium decreased the frequency of the dominant pathogens by approximately 20.7–33.3% compared with the control. This indicates that the consortium did not act selectively against only one pathogen, but provided a broad suppressive effect across the main components of the rhizosphere pathogen complex.

### 2.5. Root Yield Under Different Rotations and Biological Treatment

Root yield was significantly affected by both rotation background and biological treatments (Table 5). In the control, the highest yield was recorded in soybean → winter wheat → sugar beet (67.4 ± 1.2 t/ha), whereas the lowest was observed in soybean → maize → sugar beet (57.6 ± 1.0 t/ha). The winter wheat → soybean → sugar beet and maize → soybean → sugar beet rotations showed intermediate control yields of 60.1 ± 1.1 and 61.0 ± 1.1 t/ha, respectively.

AMF inoculation significantly increased yield in all rotations. The increase was 13.8% in winter wheat → soybean → sugar beet, 11.0% in soybean → winter wheat → sugar beet, 13.6% in maize → soybean → sugar beet, and 12.0% in soybean → maize → sugar beet. The antagonistic consortium also increased root yield, reaching 66.9 ± 1.2, 73.1 ± 1.3, 67.9 ± 1.2, and 63.2 ± 1.1 t/ha in the respective rotations.

The highest yields were obtained under combined inoculation. In soybean → winter wheat → sugar beet, yield reached 80.5 ± 1.5 t/ha, which was the highest value in the experiment. In winter wheat → soybean → sugar beet and maize → soybean → sugar beet, yields under combined inoculation reached 73.8 ± 1.4 and 74.1 ± 1.4 t/ha, respectively, whereas in soybean → maize → sugar beet yield reached 69.8 ± 1.3 t/ha.

The yield response can be interpreted as the result of two complementary processes. AMF likely improved root absorptive capacity and root–soil contact through extraradical hyphae, thereby increasing access to relatively immobile phosphorus and supporting a more active root system. By other side, the antagonistic consortium reduced the disease burden and stimulated microbial activity in the rhizosphere, which may have helped preserve root functionality during periods of pathogen pressure. Therefore, the higher yield under combined inoculation was probably associated not only with lower root rot severity, but also with better root architecture, nutrient acquisition, and microbial functional support.

### 2.6. Technological Quality of Roots

Quality traits followed the same general pattern as root yield (Table 6). In the control, the highest sugar content was observed in soybean → winter wheat → sugar beet (17.2 ± 0.2%), whereas the lowest was recorded in soybean → maize → sugar beet (16.6 ± 0.2%). AMF inoculation increased sugar content by 0.5–0.9 percentage points, depending on the rotation. The antagonistic consortium also improved this parameter, although generally to a lesser extent than AMF. The highest sugar content was obtained under combined inoculation, reaching 18.5 ± 0.2% in soybean → winter wheat → sugar beet and 18.1 ± 0.2% in winter wheat → soybean → sugar beet.

The content of molasses-forming substances showed the opposite trend. The highest values were recorded in the control of maize-containing rotations, reaching 20.2 ± 0.4 mg/100 g in maize → soybean → sugar beet and 21.1 ± 0.4 mg/100 g in soybean → maize → sugar beet. AMF reduced this parameter by 1.6–1.8 mg/100 g, antagonists by 1.2–1.4 mg/100 g, and combined inoculation by 2.0–2.4 mg/100 g, depending on the rotation. The lowest value was obtained in soybean → winter wheat → sugar beet under AMF + antagonists, where the concentration of molasses-forming substances decreased to 17.0 ± 0.3 mg/100 g.

### 2.7. Relative Contribution of Experimental Factors

Variance partitioning showed that rotation background was the main source of variation for disease development (38.4%) and root yield (29.8%), whereas mycorrhiza had the greatest contribution to AMF root colonization (46.3%) (Table 7). Antagonists accounted for 22.9% of the variation in disease development, confirming their substantial role in suppressing the pathogen complex. Year effects were also relevant, especially for yield (16.4%) and sugar content (14.3%). For sugar content, higher-order interactions and residual variance remained comparatively high (17.7%).

This structure of factor contribution suggests that crop rotation primarily acted through pathogen carryover disruption and the formation of different initial microbiological backgrounds. The AMF effect was most strongly expressed through mycorrhizal colonization and nutrient-cycling traits, whereas the antagonist effect was linked mainly to suppression of the pathogen complex and restructuring of microbial activity. The comparatively high contribution of year to yield and sugar content indicates that weather-driven variation modified the expression of these mechanisms, but did not remove the dominant role of rotation and biological regulation.

### 2.8. Principal Component Analysis of Integrated System Responses

**Figure 1:**
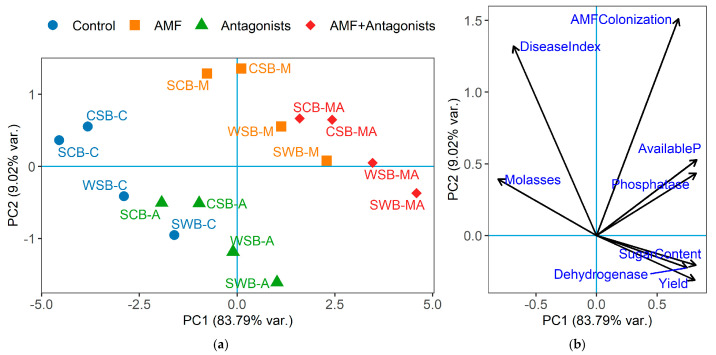
PCA biplot of the rotation × biological treatment combinations in the sugar beet experiment. WSB, winter wheat → soybean → sugar beet; SWB, soybean → winter wheat → sugar beet; CSB, maize → soybean → sugar beet; SCB, soybean → maize → sugar beet; C, control; M, arbuscular mycorrhiza; A, antagonistic consortium; MA, arbuscular mycorrhiza + antagonistic consortium. The biplot was constructed using the disease development index, AMF root colonization, available phosphorus in rhizosphere soil, dehydrogenase activity, phosphatase activity, root yield, sugar content, and molasses-forming substances. (**a**) PCA biplot of rotation and biological treatment combination; (**b**) PCA biplot of contribution and direction of analyzed variables.

To obtain an integrated view of the relationships among phytopathogenic pressure, rhizosphere functioning, root yield, and technological quality, a principal component analysis (PCA) was performed using a standardized data matrix constructed for the 16 combinations of crop rotation × biological treatment (Figure 1). The analysis included disease development index, AMF root colonization, available phosphorus in the rhizosphere soil, dehydrogenase activity, phosphatase activity, root yield, sugar content, and molasses-forming substances.

The first principal component (PC1) explained 83.79% of the total variation, whereas the second principal component (PC2) explained 9.02%. Thus, the first two components jointly accounted for 92.81% of the total variance, indicating that the two-dimensional ordination provided a highly informative representation of the system. PC1 was positively associated with available phosphorus, phosphatase activity, sugar content, root yield, dehydrogenase activity, and AMF root colonization, with loadings ranging from 0.3076 to 0.3761. In contrast, disease development index and molasses-forming substances were negatively associated with this axis, with loadings of –0.3119 and –0.3695, respectively. Therefore, PC1 can be interpreted as a general gradient of biological efficiency and functional stability: positive PC1 values reflected improved rhizosphere functioning, higher productivity, and better technological quality, whereas negative PC1 values reflected increased disease pressure and higher accumulation of molasses-forming substances.

PC2 explained a smaller but still meaningful share of the variation and reflected differences in the dominant mechanisms of plant–microbe–pathogen interaction. Positive PC2 values were primarily associated with AMF root colonization, disease development index, available phosphorus, phosphatase activity, and molasses-forming substances. In contrast, root yield, sugar content, and dehydrogenase activity were oriented toward negative PC2 values. Thus, PC2 did not represent a simple productivity gradient, but rather differentiated variants according to the balance between mycorrhizal–nutrient responses, residual disease pressure, and productive performance. In biological terms, this axis indicates that strong mycorrhizal colonization and phosphorus-related responses may occur even under conditions where phytopathogenic pressure remains relatively high, especially in less favorable rotation backgrounds.

The ordination of treatment combinations showed a clear separation between control, single-inoculant, and combined-inoculation variants. Control treatments were located mainly on the negative side of PC1, reflecting their association with higher disease development and higher contents of molasses-forming substances. This was especially evident for maize-containing rotations, where control variants occupied the least favorable ordination space. These results confirm that short rotations involving maize formed a more disease-conducive background and reduced the overall functional stability of the sugar beet rhizosphere.

Treatments with AMF or antagonists alone occupied intermediate positions between the control and the combined inoculation. AMF-treated variants were shifted toward the vectors of AMF colonization, available phosphorus, and phosphatase activity, whereas antagonistic treatments were more clearly separated from the disease index vector and more closely associated with dehydrogenase activity, indicating different functional pathways of biological regulation.

The combined AMF + antagonistic treatments formed the most favorable group and were shifted strongly toward positive PC1 values. This position indicates their integrated association with higher yield, higher sugar content, improved rhizosphere activity, and reduced negative technological indicators. The most favorable ordination space was occupied by the combined treatments in the soybean → winter wheat → sugar beet and winter wheat → soybean → sugar beet rotations, where biological inoculation was supported by a more balanced crop sequence. In contrast, combined treatments in maize-containing rotations also improved their position relative to controls, but did not fully reach the same functional state as the more favorable rotations. This confirms that biological inoculation mitigated the negative effects of short rotations but did not completely override the influence of crop sequence.

Overall, the PCA demonstrated that the response of sugar beet to crop rotation and biological inoculation was expressed as a coordinated system-level shift rather than as isolated changes in individual traits. The main gradient of variation was determined by the transition from disease-conducive, low-functioning rhizosphere conditions to biologically regulated, more productive, and technologically favorable systems. At the same time, the second component highlighted that AMF-mediated nutrient and colonization effects, antagonist-mediated pathogen suppression, and yield-quality responses were not identical processes, but complementary parts of the AMF–plant–pathogen–antagonist interaction network.

## 3. Discussion

The present study demonstrates that rotation background was the primary determinant of phytopathogenic pressure in sugar beet, with maize-containing short rotations creating the most favourable conditions for pathogen accumulation and root rot development. The maize → soybean → sugar beet sequence consistently showed the highest cumulative frequency of dominant pathogens and the highest root rot severity, whereas soybean → winter wheat → sugar beet provided the most favorable phytosanitary background. This pattern agrees with previous field evidence showing that shortened rotations do not necessarily cause an immediate decline in sugar yield, but rotations involving maize may increase the risk of *Rhizoctonia solani* crown and root rot because maize can act as a host and contribute to inoculum carryover [3,13]. The strong contribution of rotation to disease variation in the present study therefore supports the view that crop sequence acts not only as an agronomic factor, but also as a biological determinant of pathogen pressure. Additionally, our data indicate that the effect of short-rotation sequences is manifested not only through inoculum accumulation, but also through changes in the biological history of the soil, which determines the rhizosphere’s potential for mycorrhization and biological suppressiveness. A similar relationship between the preceding crop, the intensity of mycorrhization, and the productivity of the subsequent crop was reported by Arihara and Karasawa [15], who noted that non-mycorrhizal preceding crops may reduce the effectiveness of subsequent mycorrhization.

The stronger disease pressure in maize-containing rotations can be interpreted as a soil-borne legacy effect. Maize residues and the shared host range of some soil-borne pathogens, particularly *Rhizoctonia solani* AaG2-2, may maintain pathogen propagules between sugar beet crops and delay the restoration of a suppressive microbial balance. This explanation is consistent with previous sugar beet rotation studies [3] showing that shorter beet intervals do not always cause large yield losses, but maize–sugar beet sequences may increase the risk of *R. solani* crown and root rot. However, our results extend this view by showing that the negative effect of maize-containing rotations was expressed not only through yield or disease severity, but also through a broader shift in the pathogen complex and rhizosphere functioning.

An important outcome of this work is that the negative effect of short rotations was expressed at the level of the whole pathogen complex rather than through a single dominant taxon. In maize-containing rotations, the simultaneous increase in *Fusarium oxysporum*, *F. solani*, *Phoma betae*, and *R. solani* indicates that the previous crop shaped a more disease-conducive rhizosphere environment. This interpretation is consistent with current concepts of disease-suppressive soils and rhizosphere assembly, according to which plants and cropping history shape microbiome structure, microbial recruitment, and the suppressive capacity of the soil environment [16,17]. Sugar beet-specific evidence also indicates that cultivar identity, seed origin, and substrate influence rhizosphere bacterial diversity and suppression of soil-borne pathogens, emphasizing that tolerance to root diseases is partly microbiome-mediated [16]. Together, these findings support the interpretation that the observed rotation effect reflected a broader shift in rhizosphere functioning rather than a simple increase in inoculum density alone. In a broader microbiome context, these results support the modern concept of sugar beet as a holobiont system, in which root crop productivity is determined not only by genotype and agronomic background, but also by the spatiotemporal organization of the rhizosphere and endosphere microbiota [10]. Therefore, under our conditions, short rotations can be regarded as a factor destabilizing the sugar beet holobiont, whereas AMF and the antagonistic consortium may be considered tools for its partial biological reconstruction.

This interpretation is also supported by the concept of plant-driven assembly of disease-suppressive microbiomes. According to this concept [17], plants do not passively experience the soil microbiome; rather, through root exudation and immune-mediated filtering, they actively shape microbial communities in the rhizosphere. In this context, the soybean → winter wheat → sugar beet rotation likely created a more balanced microbial legacy, whereas maize-containing rotations shifted the system toward a more disease-conducive state.

AMF inoculation produced a moderate but highly consistent reduction in root rot severity across all rotations, while markedly increasing root colonization, available phosphorus, and phosphatase-related rhizosphere functioning at different crop rotation background [18]. These responses are in line with the current view of arbuscular mycorrhizal fungi as both biostimulants and biocontrol agents. At the same time, the detection of AMF colonization in the control treatment (12.8%) indicates that sugar beet roots were naturally colonized by indigenous AMF under field conditions. Therefore, the increase in colonization to 35.6% after AMF inoculation should be interpreted as reinforcement of the existing mycorrhizal background rather than as the introduction of AMF into a microbially empty system. Because native and introduced AMF taxa were not differentiated molecularly, competitive exclusion cannot be confirmed; the observed response is more consistent with functional enhancement of the indigenous mycorrhizal potential. Recent reviews indicate that AMF enhance plant performance not only by improving the acquisition of relatively immobile nutrients, especially phosphorus, but also by priming defense pathways and contributing to mycorrhiza-induced resistance [7,19,20]. Importantly, the AMF effect in our study was expressed more strongly through rhizosphere functional traits than through direct suppression of pathogen frequency, which suggests that AMF mainly improved host tolerance and rhizosphere stability. This interpretation is further supported by a recent sugar beet study showing that AMF inoculation increased root growth, phosphorus and nitrogen uptake, sugar yield, defense-related gene expression, and reduced the abundance of *Fusarium* in the rhizosphere. Previous studies have shown that AMF effects on plant diseases are context-dependent and may vary with host genotype, pathogen identity, soil phosphorus availability, native AMF communities, and environmental conditions [6,7]. Therefore, the smaller disease-suppressive effect of AMF compared with antagonists in our field experiment should not be interpreted as low biological activity, but rather as evidence that AMF primarily acted through plant-mediated and nutrient-mediated pathways under complex field conditions. Unlike the greenhouse box experiment conditions reported by Youssef et al. [11], we evaluated the mycorrhizal effect in a multi-year field experiment under different rotation backgrounds; therefore, our results further emphasize its context-dependent nature. A similar trend, although observed in table beet, was described by Yadav et al. [21], where the mycorrhizal consortium outperformed single inoculations in its effects on morphological and metabolic traits. Mechanistically, this is consistent with the findings of Hajiboland et al. [22], who showed that even active AMF mycelium can modify the antioxidant and hormonal responses of non-mycorrhizal sugar beet, indicating that the AMF effect is not limited solely to a trophic function.

The antagonistic consortium exerted a stronger direct effect on the pathogen complex than AMF alone, as reflected by the significant reduction in the frequency of all major pathogens and by the larger decrease in the root rot index. This result is consistent with the known multifunctionality of antagonistic fungi and bacteria, particularly Trichoderma-based systems, which combine competition for space and nutrients, antibiosis, mycoparasitism, induction of plant defence, and modulation of rhizosphere microbial networks [23,24]. In the present experiment, antagonist treatments also increased dehydrogenase activity, indicating intensified microbial metabolism in the rhizosphere. Similar effects have been reported in other crops, where Trichoderma reduced root rot severity while simultaneously enriching beneficial microbial groups and stabilizing rhizosphere community structure [24]. Thus, the antagonistic consortium in this study likely acted both as a biocontrol agent and as a modifier of rhizosphere activity. This functional role is important for interpreting the combined treatment, because Trichoderma can suppress soil-borne pathogens while simultaneously modifying the microbial environment in which AMF colonization is established. A similar trend was previously reported by Aly and Hussein Manal [25]: in greenhouse experiments, the combination of VAM and *Trichoderma viride* reduced the incidence of *Rhizoctonia solani* and *Fusarium solani* in sugar beet roots and improved nutrient status and quality traits. This phenomenon confirms that biological control in sugar beet production is most effective when it combines direct antagonism with the functional restructuring of the rhizosphere.

The superiority of the combined AMF + antagonists treatment indicates that the two biological components acted in a complementary rather than redundant manner. AMF primarily enhanced root colonization, phosphorus availability, and phosphatase activity, whereas the antagonistic consortium more strongly reduced pathogen frequency and increased dehydrogenase activity. The colonization level under AMF + antagonists (37.4%) was not lower than under AMF alone, indicating that the Trichoderma-based consortium did not suppress AMF establishment. This pattern supports compatibility between the introduced AMF, the antagonistic fungi, and the indigenous microbial community. Their combined application resulted in the lowest disease severity and the highest values for yield and quality-related traits. Recent literature supports the idea that combined microbial inoculation can enhance plant growth and beneficial microbial recruitment more effectively than single inoculants, although the magnitude of synergy remains context dependent and depends on host genotype, soil conditions, and inoculum compatibility [22,26]. In this case, the interaction between AMF and antagonists appears to have generated a more balanced rhizosphere environment, allowing both stronger disease suppression and more efficient resource use. This synergistic effect may be explained by the integration of AMF-mediated phosphorus acquisition and mycorrhiza-induced resistance with Trichoderma-mediated mycoparasitism, antibiosis, competition for nutrients and infection sites, and stimulation of plant defense responses. The obtained result also supports the principle of consortium-based action: functionally different microorganisms can form complementary pathways of influence on plant nutrition, protection, and the recruitment of beneficial microbiota, which is why co-inoculation often exceeds the effect of individual components [21]. Nevertheless, the response should be considered synergistic but not unlimited. The fact that maize-containing rotations remained less favorable even under combined inoculation indicates that microbial inoculants cannot completely override an unfavorable soilborne legacy. This partly explains why microbial inoculation effects often differ among studies: in greenhouse or substrate experiments, inoculants interact with a simplified microbial background, whereas in multi-year field rotations they must compete with native microbiota, fluctuating soil moisture, crop residues, and pre-existing pathogen populations [27,28].

The PCA provided an integrated confirmation of the patterns revealed by the univariate analyses. Rather than representing a set of isolated responses, the studied variables formed a coordinated functional gradient linking lower disease pressure with more active rhizosphere functioning, improved phosphorus availability, higher root yield, and better technological quality [29]. This indicates that the response of sugar beet to biological regulation should be interpreted at the system level, where phytosanitary status, microbial activity, nutrient-related traits, and production performance are tightly interconnected. In this sense, the ordination analysis supports the view that biological treatments acted not on single traits independently, but on the overall functional balance of the rotation–rhizosphere–pathogen–productivity system [27].

Equally important, the PCA helped differentiate the dominant modes of action of the two biological components. The AMF effect was more strongly aligned with traits related to root colonization, phosphorus availability, and phosphatase activity, which supports its primary role in trophic improvement and rhizosphere stabilization. By contrast, the antagonistic consortium was more closely associated with reduced disease expression and increased dehydrogenase activity, indicating a stronger contribution to pathogen suppression and microbial metabolic activation in the rhizosphere. The favorable position of the combined treatment in the ordination space therefore supports the interpretation that AMF and antagonists acted complementarily rather than redundantly, combining improved nutrient-related functioning with stronger biological control.

At the same time, the PCA confirmed that biological inoculation did not override the importance of crop rotation background. Even under biologically improved conditions, the overall system response remained dependent on the initial agroecological context created by the preceding crops [27]. This reinforces the general conclusion of the study that AMF and antagonistic microorganisms are best understood as tools for mitigating the negative consequences of shortened rotations, but not as substitutes for agronomically well-designed crop sequences. Thus, the ordination analysis strengthens the interpretation that the most effective strategy for stabilizing sugar beet production is the integration of biologically active inoculants with a phytosanitarily balanced crop rotation system.

A limitation of the present study is that pathogen identification was based mainly on cultivation and morphological criteria, while native and introduced AMF taxa were not differentiated molecularly. Therefore, some causal links between microbiome restructuring and disease suppression remain inferential. Future studies combining amplicon sequencing, quantitative pathogen assays, root exudate profiling, and functional enzyme measurements would allow a more precise separation of inoculant effects from broader rotation-induced changes in the sugar beet rhizosphere.

From an applied perspective, the observed increases in root yield and sugar content, together with the reduction in molasses-forming substances, indicate that biological regulation of the rhizosphere affected not only crop health but also technological quality. This point is especially relevant for sugar beet, where even moderate changes in sucrose accumulation and impurity content can alter processing efficiency and raw-material value. At the same time, the biological treatments did not completely eliminate the effect of crop rotation, since maize-containing rotations remained inferior to soybean → winter wheat → sugar beet even under combined inoculation. Therefore, AMF and antagonistic microorganisms should be considered tools for mitigating, rather than replacing, agronomically sound crop rotation. This conclusion is consistent with broader European assessments emphasizing crop rotation, resilience-oriented management, and biologically informed cultivation strategies as key components of sustainable sugar beet production under increasing environmental and phytosanitary pressure [1,13,30].

## 4. Materials and Methods

### 4.1. Experimental Plot Descriptions

Field experiments were conducted during 2023–2025 on the commercial fields of PE “Zakhidnyi Buh” in the Western Forest-Steppe of Ukraine (49°24′52″ N, 24°18′31″ E). The experimental plots were located on typical medium-humus chernozem soils, with some areas represented by calcareous sandy soils differing in water-holding capacity and buffering potential. The soil was characterized by 3.2–3.5% humus, 10–12 mg/100 g soil of hydrolyzable nitrogen, 12–15 mg/100 g soil of available phosphorus according to Chirikov, 18–20 mg/100 g soil of exchangeable potassium, and a slightly acidic soil reaction (pH 6.2–6.4). The climate of the study area is temperate continental. The mean annual precipitation is 580–620 mm, of which about 65% occurs during the sugar beet growing season. For the integration of multi-year data, year was included in the statistical analysis as an environmental factor. Weather conditions during the 2023–2025 growing seasons are presented in Table 8.

### 4.2. Plant Material and Experimental Design

The experimental material was the sugar beet hybrid KWS Concertina, characterized by high yield potential and good technological root quality. The field trial was established as a three-factor experiment (Table 9) in a randomized block design with four replications. The net plot area was 25 m^2^, whereas the total area of each treatment was 100 m^2^. In all crop sequences, sugar beet was grown as the final crop. Factor A was crop rotation: A1, winter wheat → soybean → sugar beet; A2, soybean → winter wheat → sugar beet; A3, maize → soybean → sugar beet; and A4, soybean → maize → sugar beet. Factor B was arbuscular mycorrhiza: B1, without inoculation; B2, AMF inoculation (*Glomus* sp.). Factor C was the antagonistic consortium: C1, without antagonists; C2, *Trichoderma erinaceum* 256 + *T. longibrachiatum* 3. Year of study (2023, 2024, and 2025) was considered an additional environmental factor.

For mycorrhizal inoculation, the product Mycofriend-tg^©^ (BTU Biotech Company, Sofiyivska Borshahivka, Ukraine) was used. It is a talc-graphite-based formulation containing sorbed arbuscular mycorrhizal fungi (*Glomus* sp., 1.0 × 10^8^ CFU/g). The product was applied simultaneously with sowing at a rate of 0.1 kg per one seed unit (100,000 seeds).

The antagonistic factor was based on two of the most active fungal isolates, *Trichoderma erinaceum* 256 and *T. longibrachiatum* 3. These isolates were selected because of their high and stable antagonistic activity against *Fusarium oxysporum*, *F. solani*, *Phoma betae*, *Rhizoctonia solani*, and *Botrytis cinerea*, as previously established in laboratory assays using the dual-culture method and qualitative signs of mycoparasitism [31]. In the field trial, the antagonistic microbial consortium was applied to the root zone on the day of sowing and again at the 4–6 leaf stage. The preparation had an initial concentration of 2.0 × 10^9^ CFU/mL and was applied at 1 L/ha after dilution in water to a final working suspension concentration of 1.0 × 10^7^ CFU/mL. The working solution was applied at a total volume of 200 L/ha using a Blu-Jet 3015 applicator.

### 4.3. Crop Management

Sowing was performed in the second decade of April, which corresponds to the optimal sowing window for the Western Forest-Steppe of Ukraine. The sowing rate was 120,000 seeds/ha. Fertilization and plant protection were kept uniform across all treatments and followed the sugar beet production technology adopted by the farm. No chemical fungicide treatments against root rots were applied during the experiment in order to avoid masking the effects of the biological factors.

### 4.4. Mycological Analysis and Assessment of Phytopathogenic Pressure

To determine the pathogen background during the growing season, root and rhizosphere soil samples were collected at canopy closure and before harvest. To characterize the pathogen background, two types of samples were collected and analyzed separately. For each crop-rotation background, 30 root samples were used for pathogen isolation from symptomatic plant tissues, whereas 20 rhizosphere soil samples were used to assess soil-associated fungal communities. The unequal number of samples reflected the different analytical purposes and methodological procedures applied to root tissues and rhizosphere soil. Surface-sterilized root fragments were plated on potato-glucose agar, whereas rhizosphere soil was analyzed using the serial dilution method. After incubation at 25–27 °C in darkness for 5–7 days, primary colonies were subcultured and identified according to their morphological and cultural characteristics. The main target pathogens were *Fusarium oxysporum*, *F. solani*, *Phoma betae*, *Rhizoctonia solani*, and *Botrytis cinerea*. The main diagnostic criteria for identification of *Fusarium* spp. are shape and size of conidia [32]; to *Phoma betae* are dark necrotic spots and formation pycnidia with unicellular conidia [33]; to *R. solani* are morphology of sclerotia [34]; to *Botritis cinerea* are grey conidial coating and multicellular oval conidia [35].

Although not every isolate was individually verified by pathogenicity testing, these fungi were repeatedly isolated from symptomatic sugar beet roots and corresponding rhizosphere soil samples in the untreated control treatments, where root rot symptoms were clearly expressed. Therefore, they were interpreted as the dominant fungi associated with the root rot complex under the studied field conditions.

### 4.5. Assessment of Root Rot Development, AMF Colonization and Rhizosphere Functioning

Root rot development was assessed using a 9-point disease severity scale followed by calculation of the disease development index (DI, %):

(1) DI (%) = (n/N) × 100

where “n” are plants showing visible symptoms of root rot; “N” are a total amount of collected plants.

In addition, the isolation frequency of each pathogen taxon (P_i_, %) and the cumulative frequency of dominant pathogens (CF, %) were calculated based on the total number of pathogen isolates recovered from the series of cultivation assays. based on Formulas (2) and (3):

(2) P_i_ = (n_i_/N_a_) × 100

where P_i_ is a frequency of isolation of the *i*-th pathogen, %; n_i_ is a number of isolates belonging to the *i*-th pathogen taxon; N_a_—total number of pathogen isolates obtained from all cultivation assays.

(3) CF = (∑n_i_/N_a_) × 100

where CF is a cumulative frequency of dominant pathogens, %; n_i_ is the number of isolates of each dominant pathogen; Na—total number of pathogen isolates.

To evaluate the effectiveness of mycorrhizal inoculation, the proportion of colonized root fragments was determined after clearing roots in 10% KOH and staining with trypan blue. Mycorrhizal colonization was expressed as the percentage of colonized root segments relative to the total number of analysed segments. Rhizosphere functioning was characterized by available phosphorus content, dehydrogenase activity, and acid phosphatase activity. Available phosphorus in rhizosphere soil was determined by the modified Chirikov method according to DSTU 4115:2002 [36]. Dehydrogenase activity was determined according to Casida et al. [37], based on the reduction of 2,3,5-triphenyltetrazolium chloride to triphenylformazan during 24 h incubation; results were expressed as mg TPF/kg soil/24 h. Phosphatase activity was determined by the method of Tabatabai and Bremner [38], based on the hydrolysis of p-nitrophenyl phosphate followed by photometric determination of released p-nitrophenol; results were expressed as mg P_2_O_5_/100 g soil/h.

### 4.6. Root Yield and Technological Quality

Root yield was determined by harvesting all roots from the net plots and converting the data to t/ha. For quality assessment, sugar content was measured by the polarimetric method, and the total content of molasses-forming substances (K, Na, and α-amino nitrogen) was expressed as mg/100 g fresh weight.

### 4.7. Statistical Analysis

Experimental data were processed by analysis of variance (ANOVA), considering the main effects of rotation, mycorrhiza, antagonistic consortium, year of study, and their interactions. Mean comparisons were performed using Tukey’s HSD_0.05_ test. For pathogen isolation frequency data, the χ^2^ test was additionally applied. In the tables, the data are presented either as means for 2023–2025 or as mean ± standard error, depending on the variable. Principal component analysis (PCA) was performed in *R* (version 4.4.3), and the ordination biplot was visualized using the *ggplot2* package (version 4.0.3).

## 5. Conclusions

Crop rotation was the key factor determining phytopathogenic pressure in sugar beet. Maize-containing short rotations increased root rot severity, whereas the soybean → winter wheat → sugar beet rotation created the most favorable phytosanitary background. AMF improved rhizosphere functioning, phosphorus availability, and enzymatic activity, while the antagonistic consortium more strongly suppressed the pathogen complex. Their combined application provided the best overall effect, reducing disease severity and improving yield and root quality. However, biological inoculants only mitigated the negative effects of shortened rotations and should therefore be used as complementary tools within integrated crop rotation-based sugar beet production systems.

## Figures and Tables

**Table 1:** Frequency of isolation of dominant pathogens (P_i_) in different rotations (source: root samples), average for 2023–2025, %.

Crop Rotation	*F. oxysporum*	*F. solani*	*Phoma betae*	*R. solani*	*B. cinerea*	Highest Cumulative Frequency of Dominant Pathogens (CF)
A1. Winter wheat → soybean → sugar beet	28.0 ± 0.8 b ^1^	17.0 ± 0.6 b	16.0 ± 0.5 b	10.0 ± 0.4 b	9.0 ± 0.3 a	80.0 ± 1.5 b
A2. Soybean → winter wheat → sugar beet	24.0 ± 0.7 c	15.0 ± 0.5 c	13.0 ± 0.4 c	8.0 ± 0.3 c	8.0 ± 0.3 a	68.0 ± 1.3 c
A3. Maize → soybean → sugar beet	33.0 ± 0.9 a	18.0 ± 0.6 a	17.0 ± 0.5 a	16.0 ± 0.5 a	10.0 ± 0.4 a	94.0 ± 1.7 a
A4. Soybean → maize → sugar beet	30.0 ± 0.8 ab	17.0 ± 0.5 ab	15.0 ± 0.5 b	14.0 ± 0.4 a	9.0 ± 0.3 a	85.0 ± 1.6 b

^1^ Within a column, means marked with different letters are significantly different according to Tukey’s HSD. Values represent the percentage of samples from which a given fungal taxon was isolated.

**Table 2:** Root rot disease severity index (DI, %) under different crop rotations and biological treatments, average for 2023–2025.

Crop Rotation	Control	AMF	Antagonistic	AMF + Antagonistic ^1^
A1. Winter wheat → soybean → sugar beet	19.2 ± 0.6 c ^2^	16.4 ± 0.5 d	14.1 ± 0.4 e	11.8 ± 0.4 f
A2. Soybean → winter wheat → sugar beet	16.5 ± 0.5 d	13.8 ± 0.4 e	11.9 ± 0.4 f	9.6 ± 0.3 g
A3. maize → soybean → sugar beet	28.6 ± 0.8 a	23.9 ± 0.7 b	20.8 ± 0.6 c	17.1 ± 0.5 d
A4. Soybean → maize → sugar beet	25.4 ± 0.7 b	21.6 ± 0.6 c	18.9 ± 0.5 cd	15.8 ± 0.5 de

^1^ This case study considers a combination of AMP inoculation and treatment with antagonistic consortia. ^2^ Means marked with different letters are significantly different according to Tukey’s HSD.

**Table 3:** AMF root colonization and rhizosphere functioning indicators, average for 2023–2025 (mean ± SE).

Treatment	AMF Root Colonization, %	Available P, mg/100 g	Soil Dehydrogenase Activity, mg TPF/kg/24 h	Phosphatase Activity, mg P_2_O_5_/100 g/h
Control	12.8 ± 0.5 b	13.2 ± 0.3 c	29.1 ± 0.7 c	4.9 ± 0.1 c
AMF	35.6 ± 0.9 a	15.0 ± 0.4 b	32.0 ± 0.8 b	5.5 ± 0.1 b
Antagonistic	14.3 ± 0.5 b	13.9 ± 0.3 c	33.4 ± 0.8 ab	5.3 ± 0.1 b
AMF + Antagonistic ^1^	37.4 ± 1.0 a	15.8 ± 0.4 a	35.8 ± 0.9 a	5.9 ± 0.1 a

^1^ Means marked with different letters in columns are significantly different according to Tukey’s HSD_0.05_.

**Table 4:** The influence of the antagonistic consortium on the frequency of isolation of major pathogens in the rhizosphere, average for 2023–2025, % (mean ± SE).

Treatment	*F. oxysporum*	*F. solani*	*Phoma betae*	*R. solani*	*B. cinerea*
Without antagonistic	29.0 ± 0.8 a	17.0 ± 0.5 a	15.0 ± 0.5 a	12.0 ± 0.4 a	9.0 ± 0.3 a
*Trichoderma erinaceum* 256 + *T. longibrachiatum* 3	23.0 ± 0.7 b	13.0 ± 0.4 b	10.0 ± 0.3 b	8.0 ± 0.3 b	6.0 ± 0.2 b
Total reduction	20.7	23.5	33.3	33.3	33.3

Within a column, means marked with different letters are significantly different according to Tukey’s HSD.

**Table 5:** Root crop yield depending on rotation and biological factors, average for 2023–2025, t/ha (mean ± SE).

Crop Rotation	Control	AMF	Antagonistic	AMF + Antagonistic ^1^
A1. Winter wheat → soybean → sugar beet	60.1 ± 1.1 e	68.4 ± 1.3 c	66.9 ± 1.2 cd	73.8 ± 1.4 b
A2. Soybean → winter wheat → sugar beet	67.4 ± 1.2 cd	74.8 ± 1.4 ab	73.1 ± 1.3 b	80.5 ± 1.5 a
A3. maize → soybean → sugar beet	61.0 ± 1.1 e	69.3 ± 1.3 c	67.9 ± 1.2 cd	74.1 ± 1.4 b
A4. Soybean → maize → sugar beet	57.6 ± 1.0 f	64.5 ± 1.2 d	63.2 ± 1.1 de	69.8 ± 1.3 c

^1^ Means marked with different letters are significantly different according to Tukey’s HSD.

**Table 6:** Quality indicators of sugar beet root crops, average for 2023–2025 (mean ± SE).

Crop Rotation	Treatment	Sugar Content, %	Molasses-Forming Substances, mg/100 g
A1. Winter wheat → soybean → sugar beet	Control	16.8 ± 0.2 de	19.6 ± 0.4 bc
	AMF	17.7 ± 0.2 bc	18.0 ± 0.3 de
	Antagonistic	17.4 ± 0.2 cd	18.4 ± 0.3 d
	AMF + Antagonistic	18.1 ± 0.2 ab	17.3 ± 0.3 e
A2. Soybean → winter wheat → sugar beet	Control	17.2 ± 0.2 cd	19.0 ± 0.4 c
	AMF	18.1 ± 0.2 ab	17.6 ± 0.3 de
	Antagonistic	17.8 ± 0.2 bc	18.0 ± 0.3 de
	AMF + Antagonistic	18.5 ± 0.2 a	17.0 ± 0.3 e
A3. maize → soybean → sugar beet	Control	16.9 ± 0.2 de	20.2 ± 0.4 b
	AMF	17.5 ± 0.2 cd	18.6 ± 0.3 cd
	Antagonistic	17.3 ± 0.2 cd	18.9 ± 0.3 c
	AMF + Antagonistic	17.8 ± 0.2 bc	18.0 ± 0.3 de
A4. Soybean → maize → sugar beet	Control	16.6 ± 0.2 e	21.1 ± 0.4 a
	AMF	17.1 ± 0.2 cd	19.3 ± 0.4 bc
	Antagonistic	16.9 ± 0.2 de	19.8 ± 0.4 b
	AMF + Antagonistic	17.5 ± 0.2 cd	18.7 ± 0.3 cd

Within a column, means marked with different letters are significantly different according to Tukey’s HSD.

**Table 7:** Dispersive contribution of factors to the variability of the main traits, %.

Source of Variation	Disease Development Index	AMF Colonization	Root Yield	Sugar Content
Crop rotation (A)	38.4	12.6	29.8	21.1
Arbuscular mycorrhiza (B)	14.7	46.3	18.5	19.2
Antagonistic consortium (C)	22.9	4.1	11.8	9.4
Year of research (Y)	12.8	8.7	16.4	14.3
A × B	3.6	9.4	6.1	7.0
A × C	4.9	2.3	5.8	4.6
B × C	1.8	8.9	5.4	6.7
A × B × C + error	0.9	7.7	6.2	17.7

**Table 8:** Weather conditions at plot.

Year	Parameter	April	May	June	July	August	September	Characteristic of Year
2023	T_air_, °C	9.8	14.9	18.2	20.3	19.8	15.1	Moderately humid; favorable for leaf canopy formation
	P_m_, mm	42	61	88	79	54	47	
2024	T_air_, °C	11.2	15.8	19.6	22.1	21.3	16.4	Warm and relatively dry; with increased stress in July–August
	P_m_, mm	36	52	63	44	39	31	
2025	T_air_, °C	8.9	13.7	17.5	19.4	18.7	14.3	Humid year; with increased phytopathogenic pressure
	P_m_, mm	58	74	96	101	67	59	

T_air_, °C—average daily air temperature; P_m_—sum of precipitation per month, mm.

**Table 9:** Field trials design.

Factor	Factor Code	Variants
Crop rotation	A	A1. Winter wheat → soybean → sugar beet
		A2. Soybean → winter wheat → sugar beet
		A3. maize → soybean → sugar beet
		A4. Soybean → maize → sugar beet
Arbuscular mycorrhiza	B	B1. Without inoculation
		B2. Inoculation by AMF (*Glomus* sp.)
Antagonistic consortium	C	C1. Without antagonistic
		C2. *Trichoderma erinaceum* 256 + *T. longibrachiatum* 3
Year of research	Y	Y1. 2023
		Y2. 2024
		Y3. 2025

## Data Availability

The original contributions presented in the study are included in the article. Further inquiries can be directed to the corresponding author.

## Abbreviations

The following abbreviations are used in this manuscript:

AMF	Arbuscular mycorrhizal fungi
